# Deciphering the spectral collapse in two-photon Rabi model

**DOI:** 10.1038/s41598-021-90085-x

**Published:** 2021-05-20

**Authors:** C. F. Lo

**Affiliations:** Institute of Theoretical Physics and Department of Physics, The Chinese University of Hong Kong, Shatin, New Territories Hong Kong

**Keywords:** Optical physics, Quantum physics

## Abstract

In this communication, based upon a squeezed-state trial wave function, we have performed a simple variational study of the spectral collapse of the two-photon Rabi model. Our analysis indicates that the light-matter interaction and the spin-flipping effectively constitute two competing impacts upon the radiation mode. Whilst the former tries to decrease the radiation mode frequency, the latter may counteract or reinforce it, contingent upon the state of the atomic system. The light–matter interaction appears to dominate the frequency modulation as its coupling strength goes beyond the critical value, leading to the emergence of the spectral collapse. However, at the critical coupling the dominance of the light–matter interaction is not complete, and incomplete spectral collapse appears. The extent of incomplete spectral collapse is found to depend upon the energy difference between the two atomic levels as well.

## Introduction

The quantum two-photon Rabi model, which is specified by the Hamiltonian $\left( \hbar =1\right)$

1 $$\begin{aligned} H=\omega _{0}S_{z}+\omega a^{\dag }a+2\epsilon \left( a^{\dag 2}+a^{2}\right) S_{x}\ , \end{aligned}$$

represents the simplest, yet nontrivial, model of the nonlinear two-photon coupling in light-matter interaction. Owing to recent advancement in the quantum technology, the two-photon Rabi model can be realized in various experimental setups over a very wide range of coupling strength^1–11^. In particular, both the trapped-ion technology^1–3^ and the state-or-the-art circuit quantum electrodynamics technology^4,11^ are anticipated to provide the most promising platforms for realizing the two-photon Rabi model. Theoretical studies have shown that the two-photon Rabi model exhibits a counter-intuitive feature, namely the “spectral collapse”, which occurs when the coupling strength $\epsilon$ is larger than half of the frequency $\omega$ of the radiation mode, *i*.*e*. $\epsilon >\omega /2$^12–24^. That is, as the coupling strength increases, the spacing of the discrete eigenenergy levels of the two-photon Rabi model diminishes monotonically, leading to a continuum energy spectrum beyond the critical value. Unfortunately, both analytical analyses (like Braak’s G-function method^15–18^) and numerical methods (such as numerical exact diagonalization^12,20^ and those based upon spectral function and continued fraction^19^) are unable to approach the collapse point satisfactorily. The characteristic behaviour of the eigenstates at the critical coupling thus remains as a mystery until recently. Lo^24^ has rigorously shown that at the critical coupling some discrete energy levels exist below a continuum energy spectrum, and the number of these bound states available depends upon the energy difference $\omega _{0}$ between the two atomic levels. In other words, incomplete spectral collapse appears at the critical coupling.

Even though the aforementioned transition has been confirmed by various theoretical studies (both analytical and numerical), a simple physical picture providing intuitive insights for the underlying physics is still lacking. In particular, the existence of incomplete spectral collapse at the critical coupling remains as a mystery. Accordingly, it is the aim of this communication to solve the mystery via an elementary quantum mechanics approach, namely a simple variational study based upon a squeezed-state trial wave function. In spite of its simplicity, the variational study is able to demonstrate rigorously how the transition occurs as the coupling strength goes beyond the critical value. Specifically, it is shown that beyond the critical value the variational estimate of the ground-state energy is not bounded below, implying that no normalisable eigenstate exists in the Hilbert space spanned by the photon number states. Our analysis indicates that the light-matter interaction effectively tries to decrease the radiation frequency whilst the spin-flipping may counteract or reinforce it, contingent upon the state of the atomic system. These two competing impacts clearly dictate the emergence of the spectral collapse. Likewise, the same approach can be applied to investigate the spectral collapse in other generalised Rabi models such as the intensity-depedent Rabi model^25^, the two-mode two-photon Rabi model^26^, and the two-photon Rabi model with a full quadratic coupling^27^ for these models share the same SU(1,1) dynamical symmetry as the two-photon Rabi model.

## Two-photon Rabi model

As in Ng et al.^12^, we first express the Hamiltonian of the two-photon Rabi model as

2 $$\begin{aligned} H=\omega _{0}S_{z}+2\omega \left( K_{0}-\frac{1}{4}\right) +4\epsilon \left( K_{+}+K_{-}\right) S_{x}\ , \end{aligned}$$

where

3 $$\begin{aligned} K_{+}=\frac{1}{2}a^{\dagger 2}, \quad K_{-}=K_{+}^{\dagger }=\frac{1}{2} a^{2}, \quad K_{0}=\frac{1}{4}\left( 2a^{\dagger }a+1\right) \end{aligned}$$

are the three generators of the Lie algebra *SU*(1,1)^28^. The Lie algebra *SU*(1,1) is defined by the commutation relations

4 $$\begin{aligned} \left[ K_{0},K_{\pm }\right] =\pm K_{\pm }, \quad \left[ K_{-},K_{+} \right] =2K_{0}\ , \end{aligned}$$

and its Casimir operator *C* is given by

5 $$\begin{aligned} C=K_{0}^{2}-\frac{1}{2}\left( K_{+}K_{-}+K_{-}K_{+}\right) =-\frac{3}{16}\ , \end{aligned}$$

which has the eigenvalue $k\left( k-1\right)$ for a unitary irreducible representation (UIR). The parameter *k* is commonly known as the Bargmann index. For the UIR known as the positive discrete series $\mathcal{D} ^{+}\left( k\right)$^28^, the eigenstates $|m,k\rangle$ of the compact operator $K_{0}$ are defined by

6 $$\begin{aligned} K_{0}|m,k\rangle =\left( m+k\right) |m,k\rangle \end{aligned}$$

for $k>0$ and $m=0,1,2,3,\ldots$, and the operators $K_{+}$ and $K_{-}$ act as raising and lowering operators respectively, i.e.

7 $$\begin{aligned} K_{+}|m,k\rangle= & {} \sqrt{\left( m+1\right) \left( m+2k\right) }|m+1,k\rangle \end{aligned}$$

8 $$\begin{aligned} K_{-}|m,k\rangle= & {} \sqrt{m\left( m+2k-1\right) }|m-1,k\rangle. \end{aligned}$$

The corresponding *SU*(1,1) generalized coherent states $|\alpha ;k\rangle$ are given by

9 $$\begin{aligned} |\alpha ;k\rangle =\exp \left\{ \alpha K_{+}-\alpha ^{*}K_{-}\right\} |0,k\rangle =\exp \left\{ \frac{1}{2}\left( \alpha a^{\dagger 2}-\alpha ^{*}a^{2}\right) \right\} |0,k\rangle. \end{aligned}$$

It should be noted that in the single-mode bosonic realization of the Lie algebra *SU*(1,1) *k* can be equal to $\frac{1}{4}$ or $\frac{3}{4}$^28^. For $k=\frac{1}{4}$ the basis states $|m,k\rangle$ consists of the even-parity states of the bosonic mode, whereas $k=\frac{3}{4 }$ refers to the subspace of the odd-parity states. For $k=\frac{1}{4}$, the coherent state $|\alpha ;\frac{1}{4}\rangle$ is simply the well-known single-mode squeezed vacuum state with the squeezing parameter $\alpha$.

Then, to faciliate a better understanding of the two-photon Rabi model, we introduce the unitary transformation^12^

10 $$\begin{aligned} R=\exp \left\{ -i\pi \left( S_{x}-\frac{1}{2}\right) \left( K_{0}-\frac{1}{4} \right) \right\} \end{aligned},$$

to decouple the spin degree of freedom from the photon mode as follows:

11 $$\begin{aligned} \tilde{H}= & {} R^{\dag }HR \nonumber \\= & {} \omega _{0}\cos \left( \pi \left[ K_{0}-\frac{1}{4}\right] \right) S_{z}+\omega _{0}\sin \left( \pi \left[ K_{0}-\frac{1}{4}\right] \right) S_{y}+ \nonumber \\&2\omega \left( K_{0}-\frac{1}{4}\right) +2\epsilon \left( K_{+}+K_{-}\right). \end{aligned}$$

Obviously, within the subspace of the Bargmann index $k=\frac{1}{4}$ the transformed Hamiltonian is reduced to

12 $$\begin{aligned} \tilde{H}_{1/4}=\omega _{0}\cos \left( \pi \left[ K_{0}-\frac{1}{4}\right] \right) S_{z}+2\omega \left( K_{0}-\frac{1}{4}\right) +2\epsilon \left( K_{+}+K_{-}\right), \end{aligned}$$

whereas for $k=\frac{3}{4}$ we obtain

13 $$\begin{aligned} \tilde{H}_{3/4}=\omega _{0}\sin \left( \pi \left[ K_{0}-\frac{1}{4}\right] \right) S_{y}+2\omega \left( K_{0}-\frac{1}{4}\right) +2\epsilon \left( K_{+}+K_{-}\right). \end{aligned}$$

In both cases each eigenstate is simply the product state $|M\rangle |\phi _{n}\rangle$, where $|M\rangle$ is an eigenstate of the spin operator and $|\phi _{n}\rangle$ the *n*-th eigenstate of the one-body bosonic Hamiltonian:

14 $$\begin{aligned} \bar{H}=M\omega _{0}\left( -1\right) ^{K_{0}-k}+2\omega \left( K_{0}-\frac{1 }{4}\right) +2\epsilon \left( K_{+}+K_{-}\right), \end{aligned}$$

for $M=\pm \frac{1}{2}$. Hence, the Hilbert space of $\tilde{H}$ comprises four different subspaces, each of which is specified by the Bargmann index *k* and the spin quantum number *M*. It should be noted that in the special case of $\omega _{0}=0$ we can diagonalize the one-body Hamiltonian $\bar{H}$ by the unitary *SU*(1,1) displacement transformation $T=\exp \left\{ -\frac{1}{2}\tanh ^{-1}\left( 2\epsilon /\omega \right) \left( K_{+}-K_{-}\right) \right\}$ as follows^12^:

15 $$\begin{aligned} \mathcal{H}=T^{\dagger }\bar{H}T=2\tilde{\omega }K_{0}-\frac{\omega }{2}, \end{aligned}$$

where $\tilde{\omega }=\omega \sqrt{1-\left( 2\epsilon /\omega \right) ^{2}}$ . It is clear that the transformed radiation mode has a lower frequency. In other words, the light-matter interaction has the effect of generating a redshift to the radiation mode frequency. Since the function $\tanh ^{-1}\left( 2\epsilon /\omega \right)$ is well-defined for $2\epsilon /\omega <1$ only, it means that there is no unitary transformation which can diagonalize $\bar{H}$ for $2\epsilon /\omega >1$. Indeed, as pointed out by Ng et al.^12^, the system represents a simple harmonic oscillator for $2\epsilon /\omega <1$, becomes a free particle at the critical coupling, and finally turns into an inverted harmonic potential barrier for $2\epsilon /\omega >1$. Such an abrupt change in the fundamental nature of the system thus leads to the collapse of a set of discrete eigenenergy levels into a continuum energy spectrum.

## Squeezed-state trial wave function

Now we perform a variational study of the ground state in each of the four subspaces. To begin with, being motivated by the aforementioned unitary *SU*(1,1) displacement transformation, we introduce a squeezed-state trial wave function which is given by

16 $$\begin{aligned} |G\rangle =U\left( \xi \right) |0,k\rangle =\exp \left\{ \frac{1}{2}\xi \left( K_{+}-K_{-}\right) \right\} |0,k\rangle \end{aligned}$$

for some real variational parameter $\xi$. Then, by direct calculation of the expectation value of $\bar{H}$ with respect to this trial wave function, we obtain an upper bound of the ground-state energy in each of the four subspaces as follows:

17 $$\begin{aligned} E= & {} \langle G|\bar{H}|G\rangle \nonumber \\= & {} M\omega _{0}\text {sech}^{2k}\left( \xi \right) +2k\omega \left\{ \cosh \left( \xi \right) +\frac{2\epsilon }{\omega }\sinh \left( \xi \right) \right\} -\frac{\omega }{2} \nonumber \\= & {} M\omega _{0}\text {sech}^{2k}\left( \xi \right) +k\omega \left\{ \left( 1+ \frac{2\epsilon }{\omega }\right) e^{\xi }+\left( 1-\frac{2\epsilon }{\omega }\right) e^{-\xi }\right\} -\frac{\omega }{2}\ . \end{aligned}$$

Apparently, for $2\epsilon /\omega <1$ the second term of *E*, *i.e*. the term with the braces, is positive definite for all values of $\xi$ so that *E* approaches infinity as $\xi \rightarrow \pm \infty$ for $\left| M\omega _{0}\text {sech}^{2k}\left( \xi \right) \right| <\omega _{0}/2$, regardless of *k*. Thus, a minimum value of *E* is guaranteed for each subspace. For $\omega _{0}=0$, the minimum appears at $\xi =$ $-\tanh ^{-1}\left( 2\epsilon /\omega \right) \equiv \xi _{0}$. It is obvious that once the spin-flip term is turned on, the minimum moves above $\xi _{0}$ for $M=-\frac{1}{2}$ and below $\xi _{0}$ for $M=\frac{1}{2}$, regardless of *k*. That is, the spin-flipping counteracts the light-matter interaction for $M=-\frac{1}{2}$ whilst reinforcing it for $M=\frac{1}{2}$. On the other hand, for $2\epsilon /\omega >1$ the term with the braces decreases monotonically as $\xi$ approaches $-\infty$, indicating that *E* is not bounded below. This is in agreement with the observations of Ng et al.^12^ that the numerical diagonalization of $\bar{H}$ using the basis states $|m,k\rangle$ in each subspace does not give any converged results at all, implying the non-existence of bound states.

Nevertheless, at the critical coupling *E* is reduced to

18 $$\begin{aligned} E=M\omega _{0}\text {sech}^{2k}\left( \xi \right) +2k\omega e^{\xi }-\frac{ \omega }{2}\ . \end{aligned}$$

For $M=\frac{1}{2}$, it is clear that $E+\omega /2$ is positive definite for all values of $\xi$ regardless of *k* and $\omega _{0}$, and that *E* approaches its minimum value, *i*.*e*. $-\omega /2$, as $\xi \rightarrow -\infty$. This clearly indicates the occurrence of spectral collapse for these two states. On the other hand, for $M=-\frac{1}{2}$ and $k=\frac{1}{4}$, since $E+\omega /2\rightarrow 0^{-}$ as $\xi \rightarrow -\infty$ and $E\rightarrow \infty$ as $\xi \rightarrow \infty$, there must exist some finite $\xi$ at which

19 $$\begin{aligned} E+\frac{\omega }{2}=-\frac{\omega _{0}}{2}\sqrt{\text {sech}\left( \xi \right) }+\frac{\omega }{2}e^{\xi }=0\Leftrightarrow e^{3\xi }+e^{\xi }-2\left( \frac{\omega _{0}}{\omega }\right) ^{2}=0\ . \end{aligned}$$

In fact, the cubic equation has one real and two complex roots^29^. Hence, according to Rolle’s theorem^29^, there must be a minimum of *E* for some finite $\xi$, implying that we always have a normalizable ground state in this subspace. Likewise, for $M=-\frac{ 1}{2}$ and $k=\frac{3}{4}$, we have $E+\omega /2\rightarrow 0^{+}$ as $\xi \rightarrow -\infty$, $E=\omega -\omega _{0}/2$ at $\xi =0$ and $E\rightarrow \infty$ as $\xi \rightarrow \infty$ so that the existence of a minimum of *E* at some finite $\xi$ is contingent upon the value of $\omega _{0}$. Specifically, if there exists some interval of finite values of $\xi$ in which $E+\omega /2<0$, then a minimum can be found at some finite $\xi$; otherwise there is none. The existence of such an interval obviously depends upon the value of $\omega _{0}$. In other words, the final outcome depends upon the competing impacts of the light-matter interaction and the spin-flipping. Simple numerical calculations show that a bound state emerges for $\omega _{0}\gtrapprox 2.1\omega$ only. This vividly illustrates how the extent of incomplete spectral collapse at the critical coupling depends upon the energy difference between the two atomic levels.

**Table 1:** Energies of the ground state and first excited state for different values of the model parameter $\omega _{0}$ are presented.

$\omega _{0}$	Upper bound of ground-state energy	Exact value of ground-state energy	Upper bound of first-excited-state energy	Exact value of first-excited-state energy
1.6	− 0.967129	− 0.987770	–	–
1.7	− 1.010847	− 1.030848	–	− 0.520494
1.8	− 1.055069	− 1.074630	–	− 0.546043
1.9	− 1.099733	− 1.118708	–	− 0.572388
2.0	− 1.144787	− 1.163174	–	− 0.599700
2.1	− 1.190188	− 1.208096	− 0.504871	− 0.628141
2.2	− 1.235898	− 1.253127	− 0.538158	− 0.657607
2.3	− 1.281885	− 1.298749	− 0.572924	− 0.687510
2.4	− 1.328122	− 1.344512	− 0.608936	− 0.719180

Finally, in order to examine the accuracy of the variational estimates, we apply the shooting method (together with the fourth-order Runge–Kutta method) to numerically solve the time-independent Schrödinger equation in Eq. (23) of Ref.^24^ for both the ground state (corresponding to $k=\frac{1}{ 4}$ and $M=-\frac{1}{2}$) and first excited state (corresponding to $k=\frac{ 3}{4}$ and $M=-\frac{1}{2}$) at the critical coupling. In Table 1 both the (numerically) exact values and variational upper bounds of the eigenenergies of these two states for different values of the model parameter $\omega _{0}$ are tabulated. Evidently, not only are the upper bounds of the ground-state energy very tight but also those of the first-excited-state energy are satisfactory. In addition, the exact calculations find that the emergence of the first excited state occurs at $\omega _{0}=1.7\omega$, which is fairly close to the variational estimate, i.e. $\omega _{0}=2.1\omega$.

## Conclusion

In this communication, by performing a simple variational study of the ground states in the four subspaces of the two-photon Rabi model, we have succeeded in demonstrating how the spectral collapse occurs as the light-matter coupling strength goes beyond the critical value. Based upon the squeezed-state trial wave function, our analysis indicates that the light-matter interaction and the spin-flipping effectively constitute two competing impacts upon the radiation mode; the former tries to decrease the radiation mode frequency whilst the latter may counteract or reinforce it, contingent upon the state of the atomic system. It is apparent that the light–matter interaction appears to dominate the frequency modulation as its coupling strength goes beyond the critical value, and this results in the emergence of the spectral collapse. However, at the critical coupling the dominance of the light–matter interaction is not complete, and incomplete spectral collapse appears. The extent of incomplete spectral collapse is also found to depend upon the energy difference between the two atomic levels, as illustrated by numerical examples. Furthermore, we believe that the same approach can be applied to investigate the spectral collapse of the intensity-dependent Rabi model^25^, the two-mode two-photon Rabi model^26^, and the two-photon Rabi model with a full quadratic coupling^27^ for these models share the same *SU*(1,1) dynamical symmetry as the two-photon Rabi model.

## References

[1] Felicetti S (2015). Spectral collapse via two-photon interactions in trapped ions. Phys. Rev. A.

[2] Puebla R, Hwang MJ, Casanova J, Plenio MB (2017). Protected ultrastrong coupling regime of the two-photon quantum Rabi model with trapped ions. Phys. Rev. A.

[3] Cheng XH (2018). Nonlinear quantum Rabi model in trapped ions. Phys. Rev. A.

[4] Felicetti S (2018). Two-photon quantum Rabi model with superconducting circuits. Phys. Rev. A.

[5] Brune M (1987). Realization of a two-photon maser oscillator. Phys. Rev. Lett..

[6] Bertet P (2002). Generating and probing a two-photon Fock state with a single atom in a cavity. Phys. Rev. Lett..

[7] Stufler S (2006). Two-photon Rabi oscillations in a single $$ In_{x}Ga_{1-x}As/GaAs$$ quantum dot. Phys. Rev. B.

[8] Del Valle E (2010). Two-photon lasing by a single quantum dot in a high-Q microcavity. Phys. Rev. B.

[9] Verma JK, Pathak PK (2016). Highly efficient two-photon generation from a coherently pumped quantum dot embedded in a microcavity. Phys. Rev. B.

[10] Qian C (2018). Two-photon Rabi splitting in a coupled system of a nanocavity and exciton complexes. Phys. Rev. Lett..

[11] Felicetti S, Hwang MJ, Boité AL (2018). Ultrastrong coupling regime of non-dipolar light-matter interactions. Phys. Rev. A.

[12] Ng KM, Lo CF, Liu KL (1999). Exact eigenstates of the two-photon Jaynes–Cummings model with the counter-rotating term. Eur. Phys. J. D.

[13] Ng KM, Lo CF, Liu KL, Lim SC, Abd-Shukor R, Kwek KH (1998). Exact dynamics of the multiphoton Jaynes–Cummings model without the rotating-wave approximation. Proc. International Conference on Frontiers in Quantum Physics, July 9–11, 1997.

[14] Emary C, Bishop RF (2002). Exact isolated solutions for the two-photon quantum Rabi model. J. Phys. A Math. Gen..

[15] Travěnec I (2012). Solvability of the two-photon Rabi Hamiltonian. Phys. Rev. A.

[16] Maciejewski AJ, Przybylska M, Stachowiak T (2015). Comment on “Solvability of the two-photon Rabi Hamiltonian”. Phys. Rev. A.

[17] Travěnec I (2015). Reply to comment on “Solvability of the two-photon Rabi Hamiltonian”. Phys. Rev. A.

[18] Duan L, Xie YF, Braak D, Chen QH (2016). Two-photon Rabi model: Analytic solutions and spectral collapse. J. Phys. A Math. Theor..

[19] Lupo E (2019). A continued fraction based approach for the two-photon quantum Rabi model. Sci. Rep..

[20] Cong L (2019). Polaron picture of the two-photon quantum Rabi model. Phys. Rev. A.

[21] Hu X (2019). The phase transition in two-photon Rabi model under mean field approximation. Int. J. Theor. Phys..

[22] Yan Z, Yao X (2020). Analytic solutions of two-photon Rabi model based on Bargmann space. IOP Conf. Ser. Mater. Sci. Eng..

[23] Armenta Rico RJ, Maldonado-Villamizar FH, Rodriguez-Lara BM (2020). Spectral collapse in the two-photon quantum Rabi model. Phys. Rev. A.

[24] Lo CF (2020). Demystifying the spectral collapse in two-photon Rabi model. Sci. Rep..

[25] Ng KM, Lo CF, Liu KL (2000). Exact eigenstates of the intensity-dependent Jaynes–Cummngs model with the counter-rotating term. Physica A.

[26] Ng KM, Lo CF, Liu KL, Lim SC, Abd-Shukor R, Kwek KH (1998). Exact dynamics of the two-mode two-photon Jaynes–Cummings model without the rotating-wave approximation. Proc. International Conference on Frontiers in Quantum Physics, July 9–11, 1997.

[27] Lo CF (2020). Manipulating the spectral collapse in two-photon Rabi model. Sci. Rep..

[28] Perelomov AM (1986). Generalized Coherent State and its Applications.

[29] Tranter CJ (1977). Techniques of Mathematical Analysis.

